# COVID-Induced Spontaneous Pneumothoraxes: Case Series

**DOI:** 10.7759/cureus.14567

**Published:** 2021-04-19

**Authors:** Sherif Elkattawy, Sarah Ayad, Islam Younes, Zamir Singh, Ramez Alyacoub, Michael L Brescia

**Affiliations:** 1 Internal Medicine, Rutgers New Jersey Medical School/Trinitas Regional Medical Center, Elizabeth, USA; 2 Internal Medicine, St. George's University School of Medicine, Elizabeth, USA; 3 Critical Care, Trinitas Regional Medical Center, Elizabeth, USA

**Keywords:** pneumothorax, pleural blebs, covid-19

## Abstract

Severe acute respiratory syndrome-related coronavirus 2 (SARS-CoV-2) is a communicable disease leading to COVID-19 infection that resulted in worldwide flooding of medical centers with the shortage of ventilators in some areas. The respiratory system is the most affected by the novel virus. Clinical manifestations are diverse in severity, with the most common symptoms including fever, chills, cough, and shortness of breath. The contributing factor to the morbidity and mortality associated with this virus is the rapid clinical deterioration as a result of a heightened inflammatory response, requiring supplemental oxygen. Pneumothorax is an unusual complication that may further worsen the hypoxia and require immediate intervention. We present a case series of two patients with no risk factors for pneumothorax besides recent COVID-19 infection, who were found to have spontaneous pneumothoraxes.

## Introduction

Severe acute respiratory syndrome coronavirus 2 (SARS-CoV-2) causing COVID-19 pandemic has led to worldwide turmoil ever since December 2019. The infections started in Wuhan, China, and extended globally thereafter. It is known to be a communicable disease, with bats being the most likely zoonotic source [1]. This novel virus has had general health and economic implications globally. Multiple organ systems have been directly affected by the novel virus, including but not limited to renal, cardiac, gastrointestinal, and respiratory systems.

In this case series, we will primarily focus on the respiratory system. As a well-documented finding of SARS-CoV-2, bilateral pneumonia is the main finding in hospitalized patients with ground-glass opacities on chest CT scan [2]. However, in this case series, we will discuss an unusual complication of COVID-19 infection: spontaneous pneumothorax.

We report a case series of polymerase chain reaction (PCR)-confirmed COVID-19 in two patients with no risk factors for pneumothorax (no smoking history, average height, no underlying lung pathology) who developed spontaneous pneumothoraxes.

## Case presentation

Case 1 presentation

**Figure 1:**
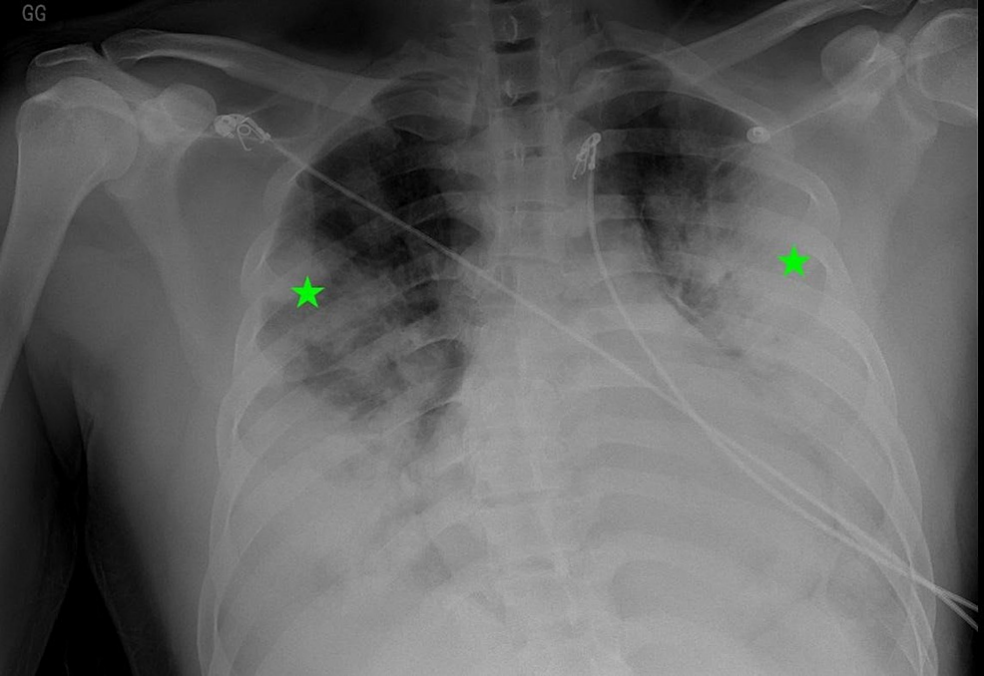
Chest x-ray showing extensive dense bilateral peripheral predominant consolidation with relative upper lung-sparing.

**Figure 2:**
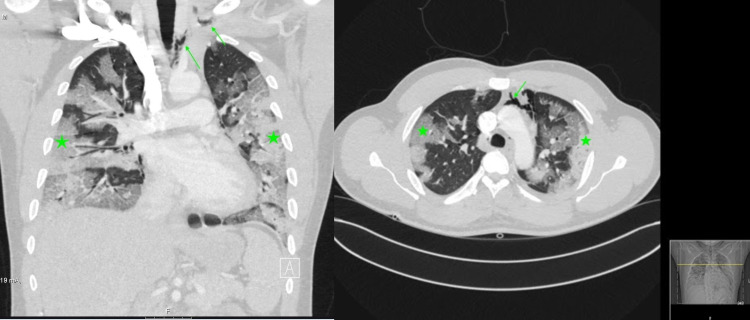
Computed tomography angiography (CTA) of the chest showing evidence of pneumomediastinum (arrow) and diffusely abnormal lungs with extensive peripheral and central ground-glass and consolidative opacities (stars).

A 26-year-old male with no significant past medical history presented to the emergency department with worsening shortness of breath for a one-week duration associated with subjective fever and cough with white sputum. He was tested positive for COVID-19 approximately one week prior to the presentation. He otherwise denied any chest pain, dizziness, lightheadedness, palpitations, or any other significant symptoms. His vital signs showed oxygen saturation of 50% on room air, respiratory rate of 39 breaths per minute, heart rate of 122 beats per minute, temperature of 98.4°F, and blood pressure at 119/99 mmHg. Physical exam was remarkable for bilateral diffuse rales. His blood work was remarkable only for elevated D-dimer 1285 (0-230 ng/ml) and fibrinogen 614 (270-500 mg/dl). Chest x-ray showed extensive dense bilateral consolidation with relative upper lung-sparing (Figure 1). Computed tomography angiography of the chest showed evidence of pneumomediastinum and diffusely abnormal lungs with extensive peripheral and central ground-glass and consolidative opacities (Figure 2). He was admitted on oxygen supplement, dexamethasone, and remdesivir. He then improved and was discharged home after nine days of hospitalization.

Case 2 presentation

**Figure 3:**
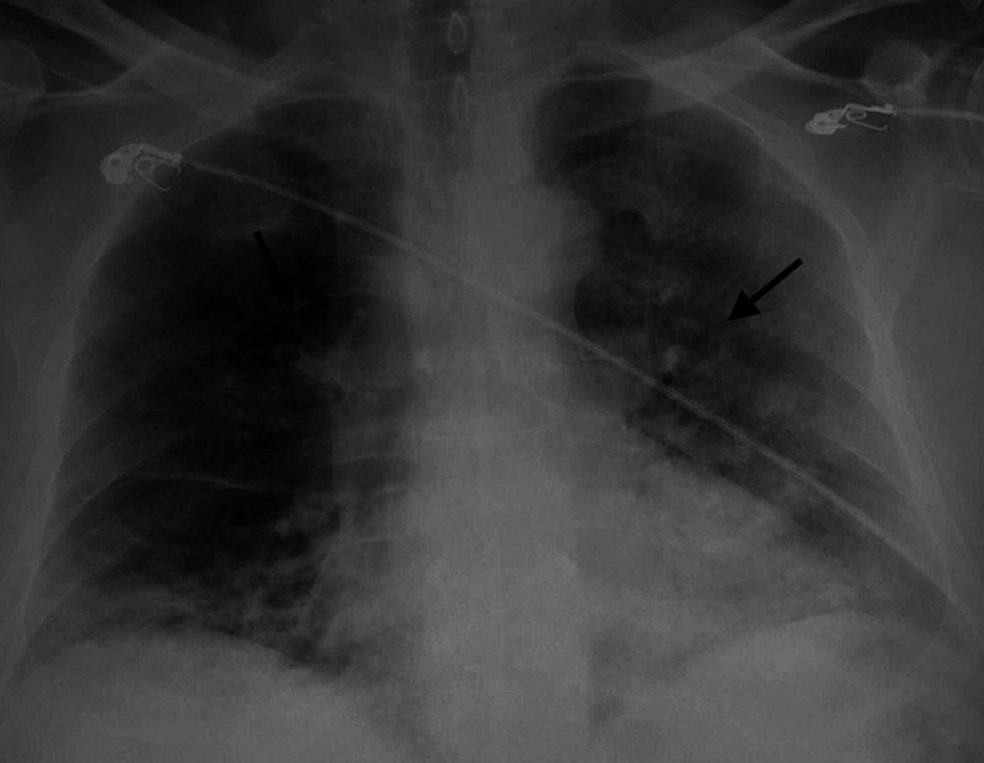
Chest x-ray shows bilateral infiltrates as shown by arrow.

**Figure 4:**
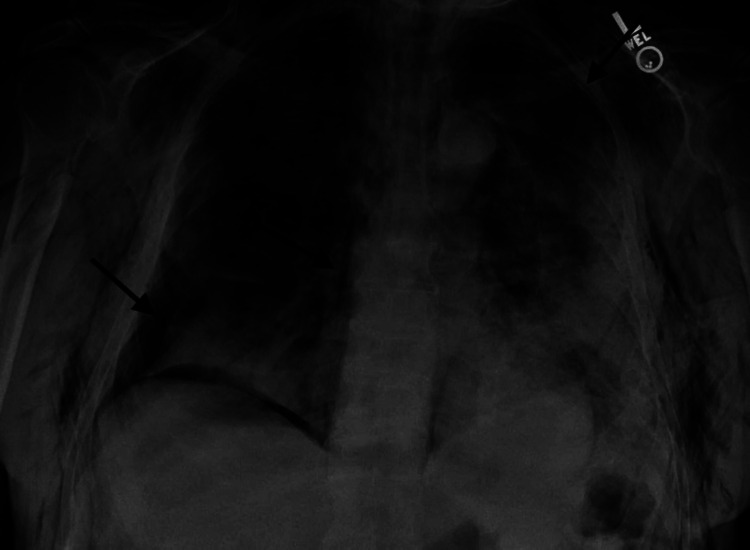
Chest x-ray shows bilateral pneumothoraces as well as pneumomediastinum as shown by arrows.

A 67-year-old Hispanic male with a past medical history of hypertension presented with a one-week history of fever, cough, generalized malaise, and shortness of breath. The patient denied any tobacco smoking history. Vital signs showed a temperature of 98.4°F, SpO2 of 85%, respiratory rate of 24 breaths per minute, blood pressure of 137/80 mmHg, and heart rate of 75 beats per minute. Lung examination was significant for scattered bilateral crackles. Chest x-ray showed bilateral infiltrates as seen in Figure 3. Labs were unremarkable except for WBC 7.8 K/uL (4.8-10.8 K/uL) with mild lymphopenia. The patient tested positive for COVID-19 and then was admitted on oxygen support with a nasal cannula, dexamethasone, remdesivir, and prophylactic enoxaparin. He initially showed improvement regarding his oxygen saturation and respiratory rate. However, he was found to be acutely deteriorating, with subcutaneous crepitations noted on his neck and anterior bilateral chest. Repeated chest x-ray showed bilateral pneumothorax, more so on the right, and subcutaneous emphysema in the upper chest and neck (Figure 4). A right chest tube was put and then transferred to the intensive care unit stay. However, his ICU course was complicated by worsening respiratory status requiring invasive mechanical ventilation and later on had a cardiac arrest secondary to COVID-19-induced acute respiratory distress syndrome (ARDS).

## Discussion

Spontaneous pneumothorax has been reported in SARS-COVID-19 pneumonia patients. Pneumothorax refers to the presence of air within the chest cavity, and it can be classified as spontaneous or traumatic pneumothorax [3]. Traumatic pneumothorax occurs secondary to trauma, including iatrogenic trauma as during central line insertion. Spontaneous pneumothorax is further subclassified as either primary or secondary. A primary pneumothorax occurs in the absence of any notable lung disease and without an apparent cause. A secondary pneumothorax occurs secondary to an existing lung or chest pathology [4].

Although the pathophysiology of pneumothorax in COVID-19 pneumonia is unclear, our case series, besides the other reported cases, support the causality between the two pathologies [4]. The literature has reported that pre-existing pulmonary cysts or pneumatoceles can be a contributing factor in such patients, especially in those who need positive pressure ventilation [4,5]. Although we do not have a prior CT chest imaging for our two cases to exclude pre-existing cyst or pleural blebs, our patient's history did not show any evidence or suspicion for such conditions. Additionally, in case 1 in our series, positive pressure ventilation was not required. In case 2, positive pressure ventilation was required after the patient deteriorated secondary to the spontaneous pneumothorax. Another possible factor is elevated intrathoracic pressure from the persistent cough leading to rupture of peripheral inflamed alveoli into the pleural cavity leading to pneumothorax [6,7]. Pulmonary embolism with subsequent pulmonary infarction and cavity formation can also be a contributing factor by rupturing of this cavity into the pleural space [8]. Our patients did not show any clinical or radiologic evidence of pulmonary embolism or infarction.

Management of such conditions is challenging. Chest tube drainage may be required whether the pneumothorax is spontaneous or secondary to barotrauma. In mechanically ventilated patients, this will be more challenging as positive pressure ventilation can contribute to prolonged air leak secondary to barotrauma. Persistent air leak despite chest tube insertion may require surgical intervention as bullectomy and pleurodesis [3,9].

Many cardiovascular and thoracic surgery societies recommend certain precautionary measures for chest tube drainage insertion in COVID-19 patients to prevent and decrease the possibility of infection transmission [9]. The American Association for the Surgery of Trauma has recommended specific measures for the safe use of conventional water seal chest drain systems such as adding bleach to the water seal chamber, applying an inline viral filter between the chest drain system and the wall suction, applying a bag-based viral filter to the suction port, and using cable ties to secure all connections in the chest tube and drainage system [9,10].

## Conclusions

SARS-COVID-19 causes a wide range of pulmonary manifestations. Deterioration of the respiratory status of the patient could be caused by pneumothorax. These two cases represent examples of pneumothorax associated with COVID-19 pneumonia. More research needed to study risk factors and management of pneumothorax in such cases.
